# Assessing mammal population densities in response to urbanization using camera trap distance sampling

**DOI:** 10.1002/ece3.10634

**Published:** 2023-10-18

**Authors:** Zhilin Li, Xiaoyi Shi, Jiayu Lu, Xiaohang Fu, Yu Fu, Yating Cui, Lu Chen, Li'an Duo, Le Wang, Tianming Wang

**Affiliations:** ^1^ Tianjin Key Laboratory of Conservation and Utilization of Animal Diversity, College of Life Sciences Tianjin Normal University Tianjin China; ^2^ Institute of Ecological Protection and Restoration, Chinese Academy of Forestry Beijing China; ^3^ Ministry of Education Key Laboratory for Biodiversity Science and Engineering, College of Life Sciences Beijing Normal University Beijing China

**Keywords:** camera trap, distance sampling, population density, scale, urban mammals

## Abstract

Environmental filtering is deemed to play a predominant role in regulating the abundance and distribution of animals during the urbanization process. However, the current knowledge about the effects of urbanization on the population densities of terrestrial mammals is limited. In this study, we compared two invasive mammals (dogs *Canis lupus familiaris* and cats *Felis silvestris*) and three indigenous mammals (Siberian weasels *Mustela sibirica*, Amur hedgehogs *Erinaceus amurensis*, and Tolai hares *Lepus tolai*) in response to urbanization using camera trap distance sampling (CTDS) in the rural–urban landscape of Tianjin, China. We used generalized additive mixed models (GAMMs) to test the specific responses of their densities to levels of urbanization. Invasive dogs (2.63 individuals/km^2^, 95% CI: 0.91–7.62) exhibited similar density estimations to cats (2.15 individuals/km^2^, 95% CI: 1.31–3.50). Amur hedgehogs were the most abundant species (6.73 individuals/km^2^, 95% CI: 3.15–14.38), followed by Tolai hares (2.22 individuals/km^2^, 95% CI: 0.87–5.68) and Siberian weasels (2.15 individuals/km^2^, 95% CI: 1.06–4.36). The densities of cats, Siberian weasels, and Amur hedgehogs increased with the level of urbanization. The population densities of dogs and cats were only influenced by urban‐related variables, while the densities of Siberian weasels and Amur hedgehogs were influenced by both urban‐related variables and nature‐related variables. Our findings highlight that the CTDS is a suitable and promising method for wildlife surveys in rural–urban landscapes, and urban wildlife management needs to consider the integrated repercussions of urban‐ and nature‐related factors, especially the critical impacts of green space habitats at finer scales.

## Graphical abstract

First study to evaluate population densities of urban mammals using camera trap distance sampling in China. Both urban‐ and nature‐related variables regulated mammal population densities. Densities were best explained by nature‐related variables on smaller scales, while urban‐related variables determined densities on larger scales.

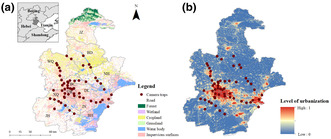

## 1 INTRODUCTION

Urbanization is tantamount to a near‐permanent alteration of land use, which eliminates the locally dominant natural ecosystem (Güneralp & Seto, 2013). It is frequently regarded as one of the most significant societal transformation processes and one of the major threats to global biodiversity (Kareiva et al., 2007). Although urban land cover constitutes only a small percentage of global land (Potere & Schneider, 2007), future urban expansion will lead to 11–33 million hectares of natural habitat loss by 2100, as well as significant natural habitat fragmentation (Li et al., 2022). Consequently, urbanization is expected to reduce local within‐site species richness by 34% and species abundance by 52% per 1‐km grid cell, with 7–9 species potentially lost per 10‐km cell (Li et al., 2022). Therefore, ecologists and managers must take decisive actions to avoid or mitigate future biodiversity loss triggered by urbanization. Knapp et al. (2021) proposed a research agenda for urban biodiversity researchers, emphasizing the importance of understanding the spatiotemporal dynamics of species and community assembly for urban wildlife conservation in the face of the sixth global extinction wave (Cowie et al., 2022). However, the current knowledge about the spatiotemporal dynamics and community assembly of urban wildlife is significantly incipient due to the low levels of biodiversity, weak species interactions, and complexities of urban ecosystems (Gallo et al., 2019).

Accumulated empirical evidence suggests that environmental filtering plays a role in determining the spatiotemporal dynamics and community assembly of urban wildlife (Aronson et al., 2016). The environmental filtering hypothesis suggests that urban species coexisting in the local pool should adapt to the surrounding biotic and abiotic environments, such as water, vegetation, food sources, and human activities, and there will be convergence and aggregation of functional traits (Gallo et al., 2017). For example, red foxes (*Vulpes vulpes*) are typical opportunistic predators exhibiting a higher occupancy probability in urban areas than less urban‐adapted bobcats (*Lynx rufus*) and coyotes (*Canis latrans*) in North America (Parsons et al., 2019). Urban‐related variables such as nighttime light, human population, and road density might also facilitate the habitat suitability of raccoon dogs (*Nyctereutes procyonoides*) in Shanghai, China (Diao et al., 2022). Density, as the basic index of the wildlife population, is vital for determining the conservation status of species, as well as for managing and making decisions about urban wildlife (Luo et al., 2020). However, due to the elusive nature, obscure behavior, smaller body size, and the limitations of statistical methods for density calculations of unmarked animals, studies assessing the environmental filtering of wildlife from the perspective of population densities have been very limited along an urbanization gradient (Howe et al., 2017).

Camera trapping (CT) is an increasingly popular and efficient method for estimating the density of elusive and rare wildlife in remote habitats (Li et al., 2019). The spatially explicit capture–recapture (SECR) approach is currently the most developed method for estimating wildlife densities accurately and precisely for individuals recognizable from images or videos (Sutherland et al., 2019). However, the majority of the species found in the urban landscape cannot be identified individually (Houa et al., 2022). In the absence of individual identification, methods for estimating densities from camera trapping data are still being developed (Burton et al., 2015). A random encounter model (REM) was created based on models predicting collision rates in an ideal gas (Rowcliffe et al., 2008). However, REM requires accurate estimates of day range and the speed of animal movement, which may be difficult to obtain or estimate accurately (Houa et al., 2022). Moreover, the accuracy and reliability of REM remain to be demonstrated (Caravaggi et al., 2016). As extensions of the REM, the random encounter and staying time (REST) model and time spent in front of the camera (TIFC) model were developed, which estimate density by using the animals' staying time in the screen as a proxy for movement speed (Becker et al., 2022). Nevertheless, all of these models have only had limited field evaluation or testing. By extending the well‐developed and widely used distance sampling framework, Howe et al. (2017) proposed camera trap distance sampling (CTDS) to estimate population density for unmarked populations (Buckland et al., 2001). CTDS enables researchers to correct for imperfect detection using a detection function to fit the decrease in detection probabilities as a function of distance between the animal and CT and then accurately calculate density (Corlatti et al., 2020). CTDS takes advantage of a consolidated mathematical framework, making the method easily accessible. Therefore, CTDS may be considered one of the most promising methods for assessing animal density, being particularly suitable for habitats where species taking advantage of dense vegetation for their cryptic existence are rarely encountered (Bessone et al., 2020). Although CTDS has received more validation than other density estimators in natural ecosystems, it is still receiving insufficient attention in wildlife management, especially in urban landscapes (Houa et al., 2022).

In this study, we applied CTDS to estimate the population densities of five dominant urban terrestrial mammals (dogs *Canis lupus familiaris*, cats *Felis silvestris*, Siberian weasels *Mustela sibirica*, Amur hedgehogs *Erinaceus amurensis*, and Tolai hares *Lepus tolai*) based on the CT monitoring network established in the rural–urban landscape of Tianjin, China. Moreover, to improve our understanding of environmental filtering of urban mammals, we examined the specific response of population densities to urban‐related and nature‐related variables. We first hypothesized that stray dog and cat densities increased with urban development, while the densities of the other three wild species showed the opposite trend. This was because urbanization might increase population density of synanthropic dog and cat by increasing available forage, or decreasing competition by reducing the population size of competitors. In contrast, urbanization can decrease population density of wild species by reducing habitat quality and quantity, increasing human disturbance, or increasing the population density of competitors (Lewis et al., 2015). Second, both natural and human‐modified habitats provide various ecological benefits to wildlife (Ramesh et al., 2017). In North America, for example, the red fox can use areas with higher urban development as spatial refuges to limit co‐occurrence with coyotes while also selecting natural habitats such as golf courses, parkland, and areas adjacent to housing (Mueller et al., 2018). Therefore, we further hypothesized that wild species densities are influenced by both urban and natural variables, whereas the densities of stray dogs and cats are influenced solely by urban variables.

## 2 MATERIALS AND METHODS

### 2.1 Study area

**FIGURE 1:**
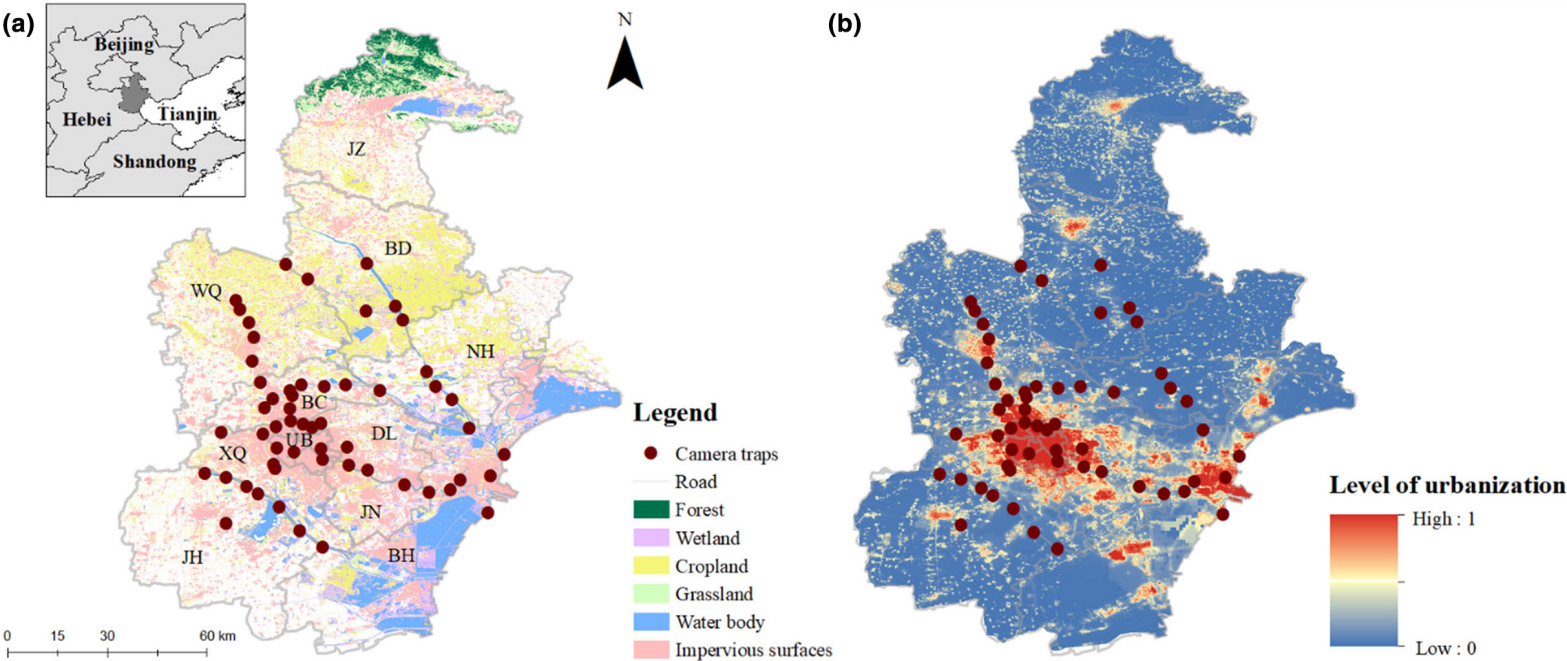
Camera locations in Tianjin, China, showing the relationship with the land types (a) and levels of urbanization (b). We classified the districts into urban areas (UB, <1000 people/km^2^), suburban areas (DL, JN, XQ, and BC, 1000–10,000 people/km^2^), and exurban areas (JH, BH, BD, NH, and WQ, >10,000 people/km^2^) based on the mean human density. Detailed descriptions of the data sources are provided in Table 1.

Tianjin (116°43′–118°4′E, 38°34′–40°15′N) is a municipality and national center city in China and is located in the northeastern North China Plain (Figure 1). Tianjin has a land area of 11,966 km^2^ and a population of approximately 14 million in 2022 (https://stats.tj.gov.cn/). As an important part of the Beijing–Tianjin–Hebei urban agglomeration, the city's GDP reached 1.57 trillion RMB (approximately 0.23 trillion USD) in 2021 (https://stats.tj.gov.cn/). Tianjin has a semihumid monsoon climate with an annual average precipitation of 665 mm and an annual mean temperature of 13°C (Zhao et al., 2016). Vascular plants occurring in Tianjin include approximately 1049 species dominated by Compositae, Gramineae, Leguminosae, and Rosaceae, such as Rowan walnut (*Juglans mandshurica*) and Amur linden (*Tilia amurensis*) (https://ghhzrzy.tj.gov.cn/). Tianjin also harbors 485 terrestrial vertebrates, including 416 bird, 43 mammal, 18 reptile, and 8 amphibian species (http://www.forestry.gov.cn).

### 2.2 Camera trap survey

We established an urban–rural landscape biodiversity monitoring network that used 56 CTs (H881, Shenzhen, China) in 14 districts of Tianjin from April to December 2021 (Figure 1). Based on the mean human density, we classified the districts into urban areas (UB, >10,000 people/km^2^), suburban areas (DL, JN, XQ, and BC, 1000–10,000 people/km^2^), and exurban areas (JH, BH, BD, NH, and WQ, <1000 people/km^2^, dominated by rural zones) (Figure 1a). All the CTs covered the majority of the urbanization levels, with 9 CTs in urban areas, 24 CTs in suburban areas, and 23 CTs in exurban areas (Figure 1b; level of urbanization ranges from 0.2 to 0.9). We used 5 × 5 km grids to guide CT placement throughout the study area. We maximized the detection probability within the sampling grids by placing cameras in urban green spaces such as parks, urban woodlands, urban wastelands, and riparian vegetation zones, which are important for urban biodiversity conservation (Aronson et al., 2017). The CTs were systematically randomly mounted at each green space patch, in accordance with the standard assumptions of distance sampling that sampling points should be placed independently of animal locations (Buckland et al., 2001). To accommodate the heights of urban mammals, the CTs were fastened on trees approximately 40–50 cm above the ground. Motion detectors were programmed to work 24 h/day and set to high sensitivity to trigger as soon as movement was detected (Cappelle et al., 2019). When triggered, the CTs recorded only 10 s of video. Each camera was visited every two or three months to download photos and check batteries.

### 2.3 Density estimations with CTDS

CTDS requires calculating the radial distance between the animal and the CT at snapshot moments (*t*) to ensure that animal movement does not bias the distribution of detection distances (Buckland et al., 2001). We thus defined a finite set of snapshot moments within the sampling period as 3 s apart (Howe et al., 2017). To estimate the distance of video‐captured individuals from the CT, we calibrated image measurements against actual measurements during camera installation (Pal et al., 2021). At each location, the CT was set to take reference photos marked by calibration poles at predefined distances from 1 to 10 m at 1‐m intervals, in the center and at each side of the camera's field of view (Cappelle et al., 2019). We also chose reference objects such as stumps and large stones to mark the distances (Harris et al., 2020). Subsequently, we recorded observation distances between the CT and the midpoint of the filmed animal (Appendix S1). For animals that traveled in groups, we treated the group as the unit of observation, measuring or estimating distances to the center of detected groups, and estimating animal density as the product of group density and group size to avoid the violation of the independence of distance observations (Buckland et al., 2001). We did not categorize the individuals by sex or age group because this was difficult to identify from the videos. Considering their smaller body size, the five mammals were assigned to 1‐m‐distance intervals ranging from 0 to 10 m. Because precise distances are more difficult to assess for objects farther away from the camera, observations >10 m were grouped together (Cappelle et al., 2019). Following exploratory analyses, we right‐truncated the data at 5 m for cats and hedgehogs, 6 m for dogs and Tolai hares, and 9 m for Siberian weasels (Appendix S2). Because the Amur hedgehogs may pass beneath the field of view (FOV), there may be a paucity of observations close to the CTs (Appendix S2). We left‐truncated the data for Amur hedgehogs at 1 m to avoid violating the assumption that detection is certain at zero distance (Howe et al., 2017).

Densities were estimated following the equation for camera trap point transects using the *distance* package in R (Howe et al., 2017; Miller, 2022):

$$\hat{D}=\frac{\sum_{k=1}^{K}n_{k}}{\pi w^{2}\sum_{k=1}^{K}e_{k}{\hat{P}}_{k}}\times \frac{1}{A}$$

where $n_{k}$ is the number of observations of animals at a point *k* (camera trap location), $e_{k}$ is the temporal effort, ${\hat{P}}_{k}$ is the estimated probability of obtaining a video of an animal that is within *θ* degrees (angle covered by the CT's field of view), *K* is the total number of CT locations, and *w* is the truncation distance beyond which any recorded distances are discarded.

The effort at a point *k* was measured as $e_{k}=\theta T_{k}/2\mathrm{\pi t}$, where θ/2π describes the fraction of a circle covered by a CT, $T_{k}$ is the period of CT deployment (in seconds), and *t* is the unit of time used to determine a finite set of snapshot moments within $T_{k}$ (Pal et al., 2021). For each CT, $T_{k}$ was calculated from 1 h after deployment until 1 h before retrieval to allow animals to become accustomed to the CTs in their environment, the smell of humans to dissipate, and to avoid any influence on the data as a result of disturbing animals while approaching a camera to recover it (Corlatti et al., 2020). CTDS obtaining unbiased density estimates relies on correspondence between the sampling period and the period describing when the target species are active and therefore available for detection (Howe et al., 2017). In this study, the weasels, hedgehogs, and hares are partly cave‐dwelling, and the cats are semiarboreal. This caused all the individuals surveyed to not be available for detection during their peak of activity; thus, the temporal sampling effort of the CTs would be overestimated, and consequently, density estimates would be negatively biased (Cappelle et al., 2019). Therefore, we calculated their activity levels (*A*) by fitting a circular normal distribution to the independent time data with a von Mises kernel using the R package *activity* and then used $\frac{1}{A}$ as the availability correction factor indicating the proportion of time when individuals of a given species are available for detection (Cappelle et al., 2019). We used a 30‐min interval between videos to consider events as independent (Wang et al., 2018).

We modeled detection functions using the key function (uniform, half‐normal, and hazard rate) plus series expansion (cosine, simple polynomial, and Hermite polynomial) approach (Howe et al., 2017). Finally, excluding the models that did not converge, we developed a suite of 13 models for the dogs, cats, and Tolai hares, 8 models for the Siberian weasels, and 10 models for the Amur hedgehogs. We selected the models following a two‐step procedure proposed by Howe et al. (2019). The best model within each key function was selected first based on the AIC adjusted for overdispersion (QAIC), where the overdispersion parameter (ĉ) was calculated from the ratio between the *χ* ^2^ statistic of the most parameterized model for each key function and its degrees of freedom (*χ* ^2^/df). Then, we compared the best models from three key functions to select the final best model with the smallest ĉ values (Table 2). The *p* values for the *χ* ^2^ goodness‐of‐fit test and the coefficient of variation (CV) were used for model evaluation, while ĉ values close to 1 indicate no evidence of significant overdispersion (Figuerola & Carlos Senar, 2007). We used the final best model to estimate the mean density of the dogs, cats, Siberian weasels, Amur hedgehogs, and Tolai hares in our study areas. Moreover, we calculated their densities in urban, suburban, and exurban areas.

### 2.4 Multiscale drivers of population densities

Urbanization is a complex process and consists of an array of components that represent the spatiotemporal changes in land type (Weng, 2007). The amalgamation of these components shapes the city's characteristic morphology and potentially determines the impacts on wildlife (Aronson et al., 2016). To test the first hypothesis, we evaluated environmental filtering by identifying the specific responses of the population densities to the five urban‐related variables (Table 1). These variables, including typical impervious surface distribution, road density, night light intensity, building distribution, and human density, represented the urbanization process in various ways (Diao et al., 2022). Furthermore, we used principal component analysis (PCA) for the urban‐related variables to generate orthogonal principal components and used the first principal component, accounting for 60% of the total variation, as a comprehensive index of urbanization level (Figure 1b) (Gallo et al., 2017). To test the second hypothesis, we examined six more nature‐related variables, including the normalized difference vegetation index (NDVI) and the distributions of cropland, grassland, forest, wetlands, and water bodies, to determine how they affected mammal densities during the urbanization process (Table 1).

**TABLE 1:** Variables used for predicting the density response of five mammal species in the green space in Tianjin, China.

	Variable name	Description	Category	Source
1	Cropland proportion	Proportion of cropland with resolutions of 30 m in 2020	Nature	GLC_FCS30‐1985_2020
2	Grassland proportion	Proportion of grassland with resolutions of 30 m in 2020	Nature	GLC_FCS30‐1985_2020
3	Forest proportion	Proportion of forest with resolutions of 30 m in 2020	Nature	GLC_FCS30‐1985_2020
4	Water body proportion	Proportion of water bodies with resolutions of 30 m in 2020	Nature	GLC_FCS30‐1985_2020
5	Wetlands proportion	Proportion of wetlands with resolutions of 30 m in 2020	Nature	GLC_FCS30‐1985_2020
6	NDVI	with resolutions of 30 m in 2021	Nature	Google Earth Engine
7	Impervious surfaces proportion	Proportion of impervious surfaces with resolutions of 30 m in 2020	Urban	GLC_FCS30‐1985_2020
8	Road density	Road density (km/km^2^) calculated from the road shapefile	Urban	OpenStreetMap
9	Night light	Distribution of night light with resolutions of 500 m in 2021	Urban	Google Earth Engine
10	Building distribution	Distribution of built‐up (BU) surfaces with resolutions of 100 m in 2020	Urban	GHS‐BUILT‐S Dataset
11	Human density	Human population densities (humans/km^2^) with resolution of 1 km in 2020	Urban	Gridded Population of the World (GPW)

To assess the scale effects of urbanization, we created concentric circles with radii ranging from 200 to 2000 m in increments of 200 m around each CT site (Lewis et al., 2015). The mean values of the variables were then calculated by the zonal statistics in ArcMap 10.7. We used the generalized additive model (GAM) to determine the best‐response spatial scales of each variable to the population densities (Li et al., 2017). We scaled and centered all continuous covariates prior to analysis to facilitate interpretations of the covariate coefficients (Wang et al., 2017). For each predictor variable, a variance inflation factor (VIF), which measures multicollinearity among variables, was calculated. Pearson's correlation coefficients were also calculated to further check for collinear variables. None of the variables in a given model were highly correlated (either Pearson's correlations > |0.5| or VIF < 3). The generalized additive mixed model (GAMM) with Gaussian‐distributed responses was used to evaluate the influence of urban‐related and nature‐related variables on the mammalian population densities using the *mgcv* package in R (Knape, 2016). The GAMM had hierarchical structures combining GAM and linear mixed‐effects models (LME) (Zuur et al., 2009). The GAM could additively evaluate the nonlinear responses of population densities to the urban‐related and nature‐related variables via smooth functions, while the LME could also do so while allowing both fixed effects (urban influence) and random effects (CT sites). Due to sample size limitations, the models had a maximum of two predictor variables and did not include interaction terms. Finally, we developed a suite of 21 models for dogs (Appendix S4), 9 models for cats (Appendix S5), 10 models for Tolai hares (Appendix S8), and 15 models for Siberian weasels and Amur hedgehogs (Appendices S6 and S7). The candidate models were then ranked using the Akaike information criterion corrected for small sample size (AICc) and their Akaike weights, with all models with ΔAICc <2 considered as competing models (Dyck et al., 2022). Model averaging and multimodel inference were conducted in the MuMIn package (Barton & Barton, 2015). We calculated Moran's I using the *morans.I* function from the R package *ape* to check the model residual for spatial autocorrelation across camera trap stations (Paradis & Schliep, 2019).

## 3 RESULTS

### 3.1 Density estimations with CTDS

**FIGURE 2:**
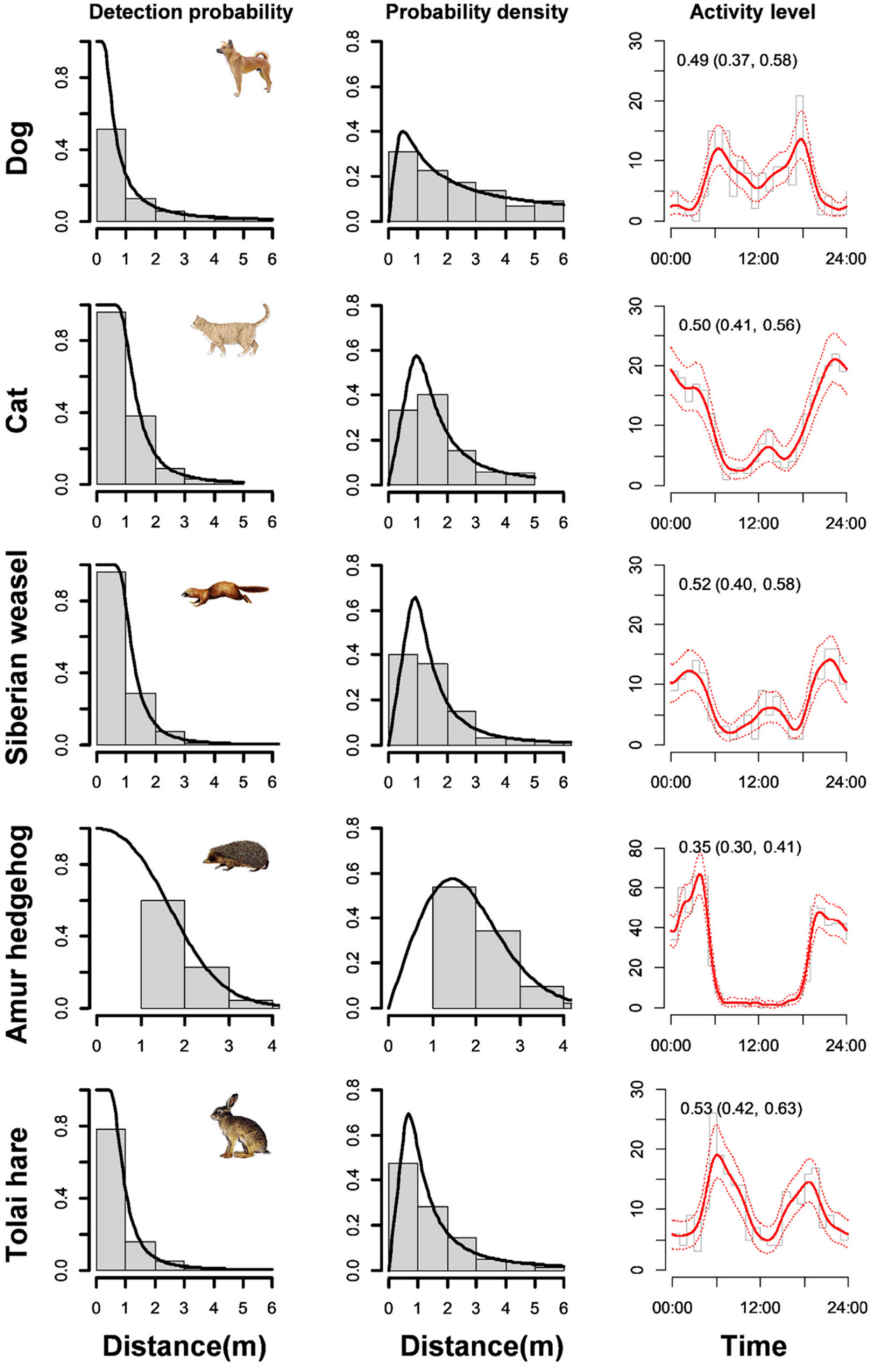
Detection probabilities as a function of observed distances between the animals and CTs from the best‐fitting CTDS of the terrestrial mammals (left column) and their probability density functions of observed distances (middle column). The daily activity levels (right column), by fitting a circular normal distribution to the time data with the von Mises kernel analysis, were used to improve density estimates.

We obtained 474 detections of dogs, 626 detections of cats, 327 detections of Siberian weasels, 616 detections of Tolai hares, and 1470 detections of Amur hedgehogs over 5776 camera days. Model selections within the key functions demonstrated that the cosine adjustments could minimize the QAIC values of uniform and half‐normal models, while the null adjustments fitted best in hazard rate models for all five mammals (Table 2). Consequently, hazard rate models with null adjustments were selected as the final best models for dog, cat, Siberian weasel, and Tolai hare, while a half‐normal model with cosine adjustments was selected for the Amur hedgehog based on the smallest ĉ values (Table 2). The detection probabilities of the mammals fitted by the final best models decreased monotonically as the distances between the animal and CTs increased (Figure 2). The cat, Siberian weasel, and Amur hedgehog exhibited nocturnal temporal activity patterns, while the dog and Tolai hare were mainly nocturnal and exhibited peaks of activity around dawn and dusk (Figure 2). The Amur hedgehog exhibited the lowest activity level of 0.35 (95% CI: 0.30–0.41), while the Tolai hare had the highest activity level of 0.53 (95% CI: 0.42–0.63).

**TABLE 2:** Details of the top three models used to estimate the densities of dogs, cats, Siberian weasels, Amur hedgehogs, and Tolai hares in the urban green space in Tianjin, China.

Key function	Adjustment type	Order	QAIC	Ĉ	χ^2^‐*p*	CV	Density (95% CI)
**Dog**							
Hazard rate	Null	0	444.61	1.25	.29	0.56	2.63 (0.91, 7.62)
Half‐normal	Cosine	2	96.15	9.45	<.01	0.40	1.11 (0.48, 2.57)
Uniform	Cosine	1,2	37.42	28.3	<.01	0.39	0.58 (0.25,1.35)
**Cat**							
Hazard rate	Null	0	308.57	0.82	.44	0.24	2.15 (1.31, 3.51)
Half‐normal	Cosine	2	39.41	3.13	.04	0.23	2.67 (1.66, 4.30)
Uniform	Cosine	1,2	768.10	29.86	<.01	0.23	1.33 (0.83, 2.14)
**Siberian weasel**							
Hazard rate	Null	0	705.4	0.96	.45	0.33	2.15 (1.06, 4.36)
Uniform	Cosine	1,2	17.79	103.69	<.01	0.32	0.37 (0.18, 0.74)
**Amur hedgehog**							
Half‐normal	Cosine	3	2283.67	0.64	.42	0.39	6.73 (3.15, 14.38)
Uniform	Cosine	1,2	1125.38	1.63	.20	0.32	6.39 (3.39,12.04)
**Tolai hare**							
Hazard rate	Null	0	320.4	1.42	.23	0.48	2.22 (0.87, 5.68)
Half‐normal	Cosine	3	83.23	24.58	<.01	0.46	2.35 (0.94, 5.83)
Uniform	Cosine	1	17.09	124.52	<.01	0.45	0.34 (0.14, 0.85)

*Note*: The table reports key functions (defining parametric shapes for the detection function), adjustment types (to allow for departures from the parametric shape), the number of adjustment terms selected (order), Akaike's information criterion adjusted for overdispersion (QAIC), overdispersion factor (Ĉ), significance of χ^2^ goodness‐of‐fit test (χ^2^‐*p*), coefficient of variance (CV), and density estimates with 95% confidence intervals.

**FIGURE 3:**
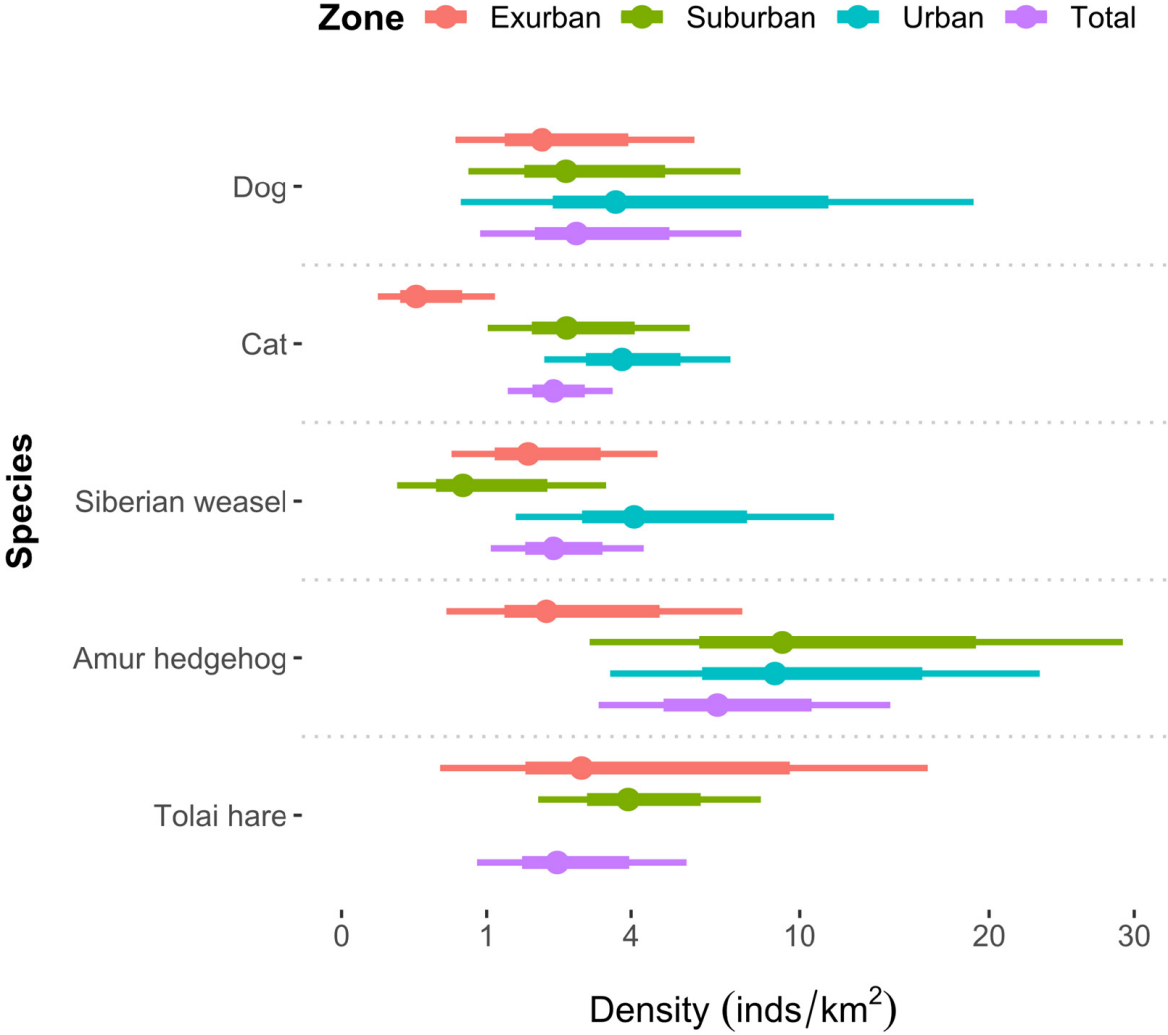
Density estimates with 95% confidence intervals for dogs, cats, Siberian weasels, Amur hedgehogs, and Tolai hares across the gradient from exurban to urban.

The Amur hedgehog had the highest density of 6.73 (95% CI: 3.15–14.38) individuals/km^2^, while the other mammals exhibited similar density estimations ranging from 2.15 to 2.63 individuals/km^2^ (Table 2; Appendix S3). Cat densities were significantly lower in exurban areas (0.26 individuals/km^2^, 95% CI: 0.06–0.12) than in suburban (2.42 individuals/km^2^, 95% CI: 1.01–5.78) and urban (3.75 individuals/km^2^, 95% CI: 1.95–7.21) areas (Figure 3). We did not detect Tolai hares in urban areas during the research period.

### 3.2 Density responses of mammals to urbanization

**FIGURE 4:**
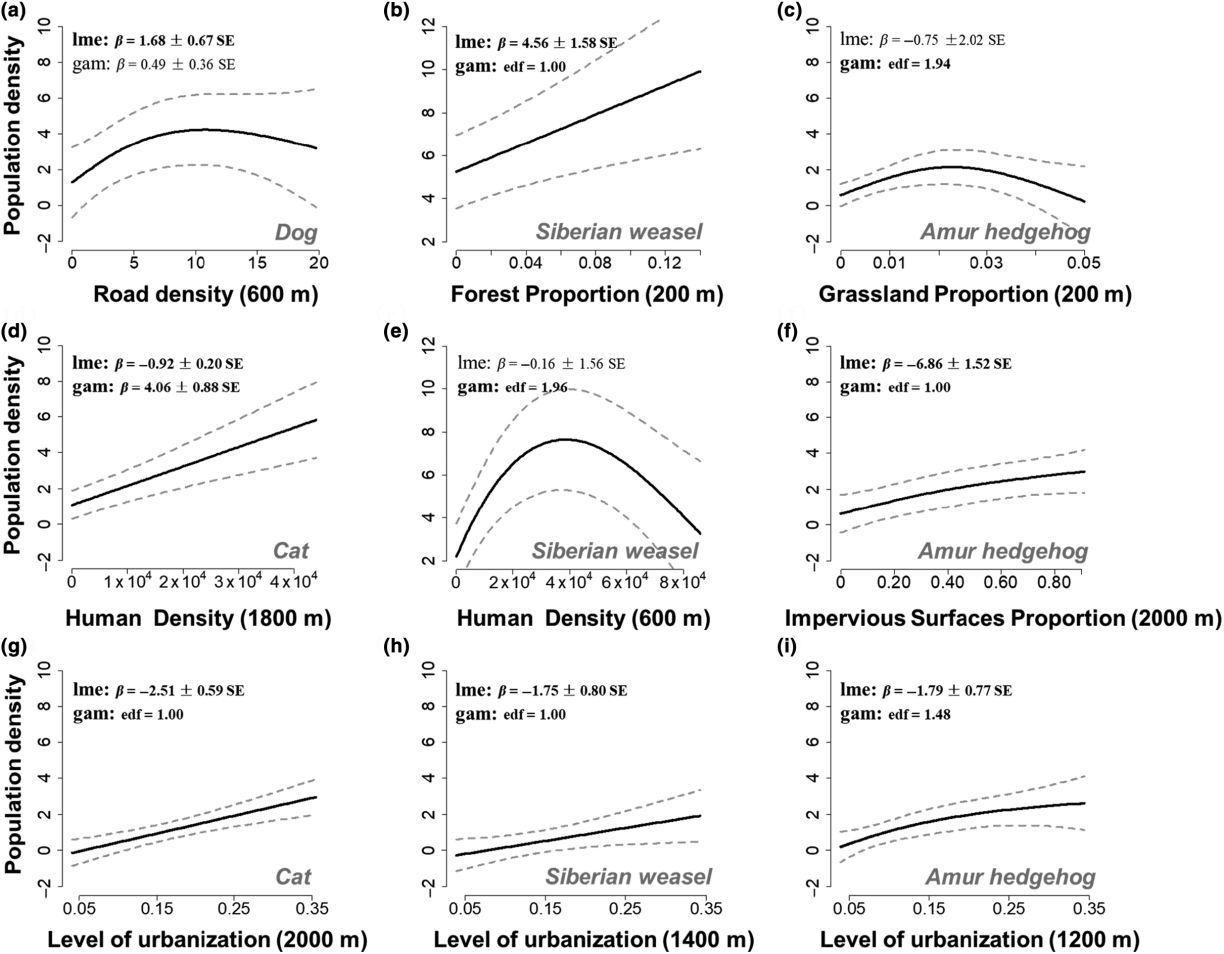
Influence of urban and natural variables on the population densities of mammals evaluated by generalized additive mixed models (GAMMs) on the best‐response scales in Tianjin. The fixed effects of GAMMs were split into parametric coefficients (LME) and smooth terms (GAM), while boldface type indicates statistical significance at the 95% level or better. The curves of Panel (a) and Panel (d) were estimated by the model averaging of multimodel inference in the MuMIn package, and the output styles of both GAM and LME were beta estimations with standard errors. The curves of Panel (b,c and e‐i) were estimated by the single top GAMM, and the output styles of GAM were edf values.

The results of the GAMM showed that dog and cat densities increased with urban development. Both the LME and GAM indicated significant positive effects of human density and urban levels on cat densities (*p* < .05 in Figure 4d,g). The LME also demonstrated that dog densities increased slowly with road density (with a buffer radius of 600 m) and peaked around the road density of 10 km/km^2^ (*β* = 1.68 ± 0.67 SE, *p* < .05 in Figure 4a). However, we did not find apparent spatial avoidance of urbanization by wild mammals. The LME and GAM indicated that the population densities of the Siberian weasel and Amur hedgehog increased with urban levels (Figure 4h,i). Specifically, the population density of the Amur hedgehog also increased with the proportion of impervious surfaces (Figure 4f), while the density of the Siberian weasel exhibited significant nonlinear correlations with human density by the GAM (edf = 1.96, *p* < .05) and peaked at approximately 4000 people/km^2^ (Figure 4e).

Our results supported the second hypothesis that the population densities of wild species are influenced by both urban‐related and nature‐related variables, while stray dogs and cats are influenced only by urban‐related variables (Figure 4). In addition, we found that densities were best explained by nature‐related variables at small scales (e.g., 200 m; Figure 4b,c), while urban‐related variables determined densities at larger scales (e.g., other panels in Figure 4). The Siberian weasel density increased with the proportion of forest with a key spatial scale of 200 m (Figure 4b) but also responded to human density with a buffer size of 600 m (Figure 4e) and a level of urbanization of 1400 m (Figure 4h). Moreover, the GAM indicated that the population density of the Amur hedgehog was also influenced and exhibited nonlinear relationships with the proportion of grassland around the CTs (200 m radius, Figure 4c). However, the LME did not show a significant relationship between grassland proportion and Amur hedgehog density (*β* = −0.75 ± 2.02 SE, *p* = .71 in Figure 4c). We did not find significant effects of urban and natural variables on the population density of the Tolai hare. The residuals were highly variable across sites and showed no spatial autocorrelation for the dogs (Moran's I *p* = .27), cats (Moran's I *p* = .10), Siberian weasels (Moran's I *p* = .78), and Tolai hares (Moran's I *p* = .32), while the model displayed significant residual autocorrelation for Amur hedgehogs (Moran's I *p* = .01).

## 4 DISCUSSION

### 4.1 Density estimations with CTDS

CTDS allows for the simultaneous monitoring of multiple unmarked species concurrently at large spatial scales (Li et al., 2022). This method has been used to estimate the density of many large mammals in their natural habitat, including western chimpanzee (*Pan troglodytes verus*), desert bighorn sheep (*Ovis canadensis*), Himalayan musk deer (*Moschus leucogaster*), and bharal (*Pseudois nayaur*) (Cappelle et al., 2019; Harris et al., 2020; Pal et al., 2021). However, the applicability of CTDS in urban–rural landscapes has never been assessed. The ability of CTDS to generate density estimates across multiple species and habitats from a single survey is encouraging, implying that the method could be deployed in multiple ecosystems, including urban landscapes (Mason et al., 2022). Compared to natural ecosystems, urban ecosystems exhibit simpler habitat structures and empty habitat patches, especially in well‐tended parks and urban woodlands (Nassauer & Raskin, 2014). This helped to maintain higher detection probabilities for mammals, decreasing the bias of density estimations. Nevertheless, there was still dense bushwood and grass in the urban wasteland and riparian vegetation zones caused by secondary succession, especially in summer. This might also influence detection probabilities and the actual angle of the CTs, eventually leading to biased estimations (Pal et al., 2021). In general, the relatively small body size and elusive activities of urban mammals limit their detection by CTs, especially in dense vegetation (Lashley et al., 2018). The smaller urban mammals also led to closer effective detection distances, which is the reason why peak probability density values for detections in our study were all approximately 1 m (Figure 2). In comparison with larger mammals in their natural habitat, small mammals barely block the view of animals that were further away. This makes calculating the distance from the camera for individuals in the background simple, avoiding a bias toward individuals recorded at shorter distances (Pal et al., 2021). Moreover, the closer detection distances led to more precise measurements of the urban mammals, and the imprecise measures of far distances should not be an issue by appropriately binning the distance in CTDS (Buckland et al., 2015).

CTDS precision was primarily determined by detection probabilities (which were influenced by the number of observation distances) and heterogeneous distribution of encounter rates (expected number of distance observations per unit effort, which were mainly influenced by the number of individual locations with at least one capture event) (Howe et al., 2017). Given that the number of observations used to model the detection process was usually large (>100) and the *χ* ^2^ goodness‐of‐fit test statistic indicated sufficient accuracy for calculation of the detection probabilities (*p* > .05, Table 2), we concluded that the variation in abundance estimates was large (i.e., dog CV = 0.56 and Tolai hare CV = 0.48 in Table 2) due to encounter rates rather than detection probability (Cappelle et al., 2021). These results partly reflected the heterogeneous distribution of mammal abundance caused by environmental filtering, while necessitating the establishment of more sampling locations to increase the precision of the population density estimations. In addition, the bias of encounter rates may be regulated by animals' complex responses to CTs, such as avoidance or attraction (Cappelle et al., 2021). If animals avoid the field of view of the CTs or remain in front of the CTs for less time than expected, the population size or density of that species may be underestimated (Houa et al., 2022). Conversely, animals that stay longer in the detection zone cause an overestimation of the population size or density. To address this issue, Bessone et al. (2020) recommend either deploying CTs for at least 1 month prior to the survey, reducing mammalian visual and olfactory reactivity to CTs, or recording reference distance markers after the survey, and reducing the CT setup time. We found no clear attraction reactions of animals to CTs in this study. This is due in part to the fact that the data were collected from April to December 2021, while the CT monitoring network was launched in 2019. This time interval allows mammals enough time to become accustomed to CTs. Furthermore, wildlife in urban areas is exposed to novel environmental pressures, including many artificial facilities in combination with chemical, acoustic, and light pollution (Grimm et al., 2008). These environmental pressures may lead to urban wildlife behaving differently than animals living in natural environments (Adams, 2018). We also found many trash objects and construction debris in urban green spaces, such as wires, packing boxes, and metal containers resembling CTs (Zhang et al., 2010). Abandoned garbage may also help to make urban animals more accustomed to CTs. Considering the low encounter rates, however, we have to extend the survey periods to 8 months to get a better fit for the detection process. This may cause the violation of population closure assumption and lead to the underestimates of detection probability and overestimates of population densities (Yamaura & Royle, 2017).

### 4.2 Scale‐dependent drivers of population densities

The results support the environmental filtering of mammalian population densities on multiple scales along rural–urban gradients in Tianjin, China. The effect of a landscape variable on a given species and for a given biological response is typically strongest at a particular spatial scale (Moll et al., 2020). Our study elucidated the difference in the best‐response scales of nature‐ and urban‐related variables for mammals. Specifically, population densities were best explained by nature‐related variables on smaller scales but by urban‐related variables on larger scales (Figure 4). This implied that the large‐scale layout planning of urbanization could impact the local population densities of mammals. Moreover, even small natural patches will also be critical to influencing mammal populations in the urban landscape (Saito & Koike, 2013). We highlight the ecological significance of small green spaces and recommend more small green spaces mosaicked within urban landscapes to maintain mammal populations. This might be conducive to relieving conflicts between urban development and biodiversity conservation, especially in response to land scarcity issues in urban landscapes (Mao et al., 2020).

Our results cannot totally support or reject the first hypothesis because, with the exception of Tolai hares, the densities of both the invasive species (dogs and cats) and native wild mammals (Siberian weasels and Amur hedgehogs) all increased with urbanization. In this study, we estimated the densities of dogs and cats without distinguishing the semi‐free‐range individuals (so‐called indoor/outdoor pets) from the stray individuals because we could not identify them through videos. However, both types of pets rely heavily on anthropogenic products such as discarded food waste, cultivated fruits and crops, and pet food (Cove et al., 2022). For example, in Brazil, stray dogs preferred leftover food offered voluntarily by restaurant patrons (Dias et al., 2013). In university campuses in China, the density of free‐ranging cats was found to be positively associated with the density of feeding stations (Li et al., 2020). In addition, dense road networks provide urban dogs with additional pathways for movement (Srbek‐Araujo & Chiarello, 2008), which may explain the positive correlation between roads and dog density.

Our study highlights that the effects of urbanization on mammal densities should be estimated using both the synthetic urbanization index and specific urban‐related variables. In this study, the spatial patterns of mammal densities may be best explained by different urban‐related variables. Siberian weasels and Amur hedgehogs were found to be typical urban adapters with higher densities in urban areas. This behavior has also been recorded in common raccoons (*Procyon lotor*), red foxes, and some birds, such as magpies (*Pica pica*) and crows (*Corvus corone*) (Hubert et al., 2011). Compared to rural areas, urban areas could provide more favorable conditions for urban adapters, including better habitat quality (Cheptou et al., 2008), higher anthropogenic food availability (Contesse et al., 2004), more suitable vegetation structures for shelter (Baker & Harris, 2007), and more beneficial climatic conditions (Pickett et al., 2001). Environmental temperature factors were identified as essential determinants of hedgehog density (Jackson, 2007). Spring/summer temperature may have a significant impact on food supply (Jackson & Green, 2000), which may determine hedgehog body condition and thus survival rate during winter because energy stored as fat is essential for survival during hibernation (Cherel et al., 1995). High year‐round food availability is also assumed to increase longevity and reproductive success in urban adaptor populations, but this assumption is not always accepted (Hubert et al., 2011). Urban adaptors often use artificial food as a supplement to their natural diet, not as a substitute (Hubert et al., 2011). Thus, Siberian weasels and Amur hedgehogs also need to select natural patches (e.g., forests, bushes, and grassland) that hold a rich abundance of natural diets, such as rodents, earthworms, and terrestrial arthropods (Hubert et al., 2011; Munshi‐South, 2012). Consequently, forest and grassland habitats were critical in determining the population densities of Siberian weasels and Amur hedgehogs in rural–urban landscapes.

Environmental filtering employs a commonly used framework in community assembly theory, that of hierarchically imposed filters through which mammals must have the appropriate traits to pass to colonize or persist in more urbanized areas (Aronson et al., 2016). Urban mammals thus need to have greater dispersal potential and abilities to adapt to the complexity of the urban landscape and tolerate high levels of anthropogenic activities. In this study, we detected limited numbers of mammal species in the urban–rural landscape of Tianjin. However, in the natural habitat of Yanshan Mountain in northern Tianjin, CT surveys revealed higher species richness, including Chinese goral (*Naemorhedus griseus*), Siberian roe deer (*Capreolus pygargus*), hog badger (*Arctonyx collaris*), masked palm civet (*Paguma larvata*), and leopard cat (*Prionailurus bengalensis*), but none of these species were detected in the rural–urban landscape (Li et al., 2018). In addition, the Tolai hare was detected only in exurban and suburban areas, not in urban areas (Figure 3). The striking differences in population density along the urbanization gradient reflected the environmental filtering of various mammals along the rural–urban gradient.

Aside from environmental filtering, pressures from intraguild predation and interspecies interactions have been suggested as key drivers of spatial distribution and population declines of urban wildlife. Moreover, the intensities and directions of the interactions were mediated by their densities (Pettett et al., 2018; Taucher et al., 2020). However, in this study, we failed to consider interactions. Stray dogs and cats have been observed preying on young or injured hedgehogs (Reeve & Huijser, 1999). Our CTs also found evidence of aggressive behaviors of the stray cat toward the Siberian weasel. The density‐mediated influence of species interactions on environmental filtering requires further attention. Due to the methodological limitations, we can only obtain the specific density estimations of each CT sites without variances using CTDS. However, the uncertainty carried over from the distance sampling may also influence the analysis of GAMMs and to some extent impact the understanding of environmental filtering. Harrell (2001) argued that the ratio of observations to predictor variables should be at least 10 or 20 to avoid over‐fitting. While we only conducted 56 CTs accounting for the effects of 11 predictor variables on densties. The implication is degraded model performance in the estimation stage even though the models had a maximum of two predictor variables in multivariable regressions. This also necessitated an adequate sample size to generate reasonably accurate estimates of variables in the future analysis.

## 5 CONCLUSION

Our study shows that the CTDS is a suitable and promising method for large‐scale wildlife monitoring in rural–urban habitats. The ability of CTDS to generate these habitat‐specific density estimates will benefit conservation management by filling data gaps and providing new insights into the effects of urbanization on wildlife. To the best of our knowledge, this is the first study in China to use CTDS to determine the precise densities of urban mammals. However, our findings can only represent their population densities in Tanjin's urban green spaces. We suggest conducting density estimations in more cities and more urban components, such as industrial, commercial, and residential areas. To gain a better understanding of urbanization effects, we recommend evaluating the abundance and occupancy of urban wildlife using both the synthetic urbanization index and specific urban‐related variables at multiple scales.

## AUTHOR CONTRIBUTIONS

**Zhilin Li:** Conceptualization (equal); data curation (lead); methodology (lead); writing – original draft (lead); writing – review and editing (lead). **Xiaoyi Shi:** Investigation (equal). **Jiayu Lu:** Data curation (equal); investigation (equal). **Xiaohang Fu:** Data curation (equal). **Yu Fu:** Data curation (equal); investigation (equal). **Yating Cui:** Data curation (equal); investigation (equal). **Lu Chen:** Data curation (equal). **Li'an Duo:** Project administration (equal). **Le Wang:** Resources (equal). **Tianming Wang:** Data curation (lead); formal analysis (lead); funding acquisition (lead); methodology (equal).

## FUNDING INFORMATION

This work was funded by the National Natural Science Foundation of China (31971539), the National Science and Technology Basic Resources Survey Program of China (2019FY101700; 2021FY1007020), and the Open Fund of Key Laboratory of Biodiversity Science and Ecological Engineering, Ministry of Education (K202304).

## CONFLICT OF INTEREST STATEMENT

We have no conflicts of interest to report for this manuscript.

## Supporting information


Appendix S1‐S8

## Data Availability

The distance data of the five terrestrial mammals for camera trap distance sampling analysis have been uploaded in Dryad at https://datadryad.org/stash/share/dN5ZezCc5goEOm‐l50ETTJJEPeUl8sjrCR45zYhvM5Y.
