# Supplementary material for: Assessing mammal population densities in response to urbanization using camera trap distance sampling
Source: Ecol Evol. 2023 Oct 18;13(10):e10634. doi: 10.1002/ece3.10634 (PMC10582676; doi:10.1002/ece3.10634)
Supplement: Supplementary file 1 — Appendix S1‐S8 [file ECE3-13-e10634-s001.docx]

**Appendix S1** Reference photos marked by calibration poles at predefined distances from 1 to 10 meters at 1‐meter intervals can help to estimate the distance of video-captured individuals from the CT.


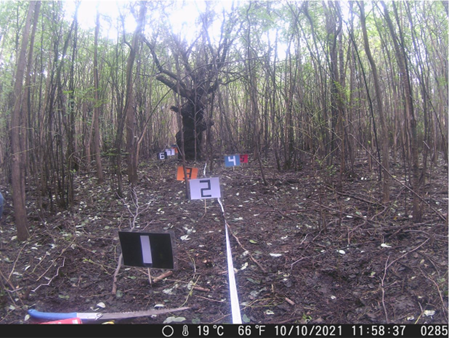

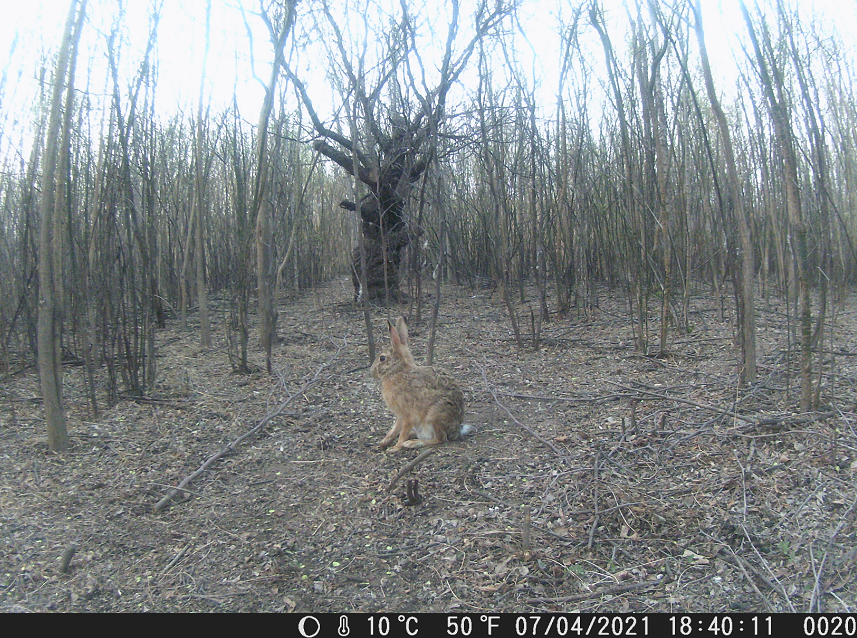


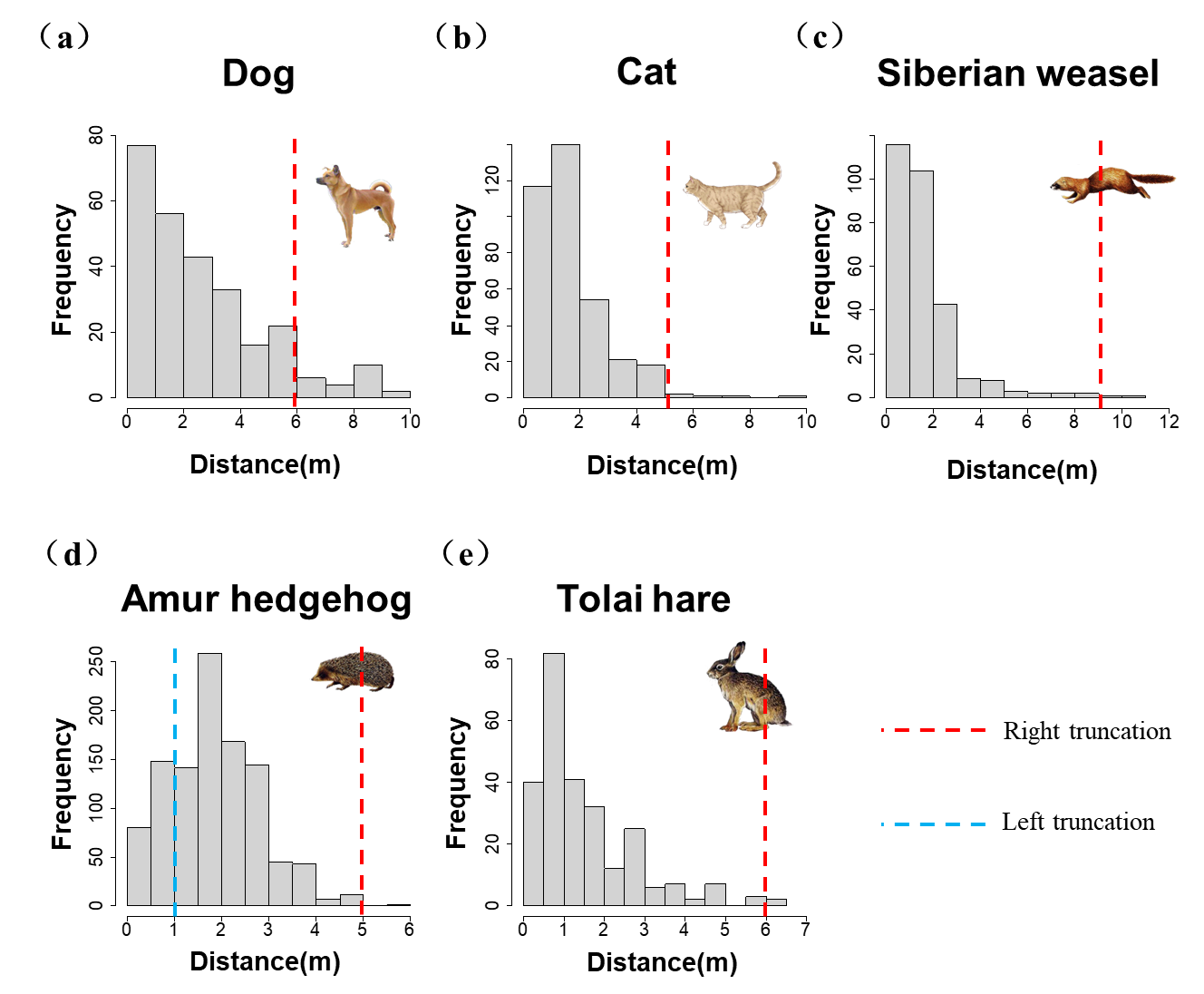


**Appendix S2** Exploratory analyses helped to identify the left-truncated and/or right-truncated distances for terrestrial mammals to avoid violations of the assumption that detection is certain at zero distance.

**Appendix S3** Density estimates with 95% confidence intervals for dogs, cats, Siberian weasels, Amur hedgehogs and Tolai hares in urban, suburban, exurban and whole study areas in Tianjin using camera trap distance sampling.

| **Species** | **Density (individuals/km^2^)** | | | |
| --- | --- | --- | --- | --- |
|  | **Exurban** | **Suburban** | **Urban** | **Total** |
| Dog | 1.91 (0.62, 5.94) | 2.41 (0.76, 7.58) | 3.59 (0.68, 19.06) | 2.64 (0.91, 7.62) |
| Cat | 0.26 (0.06, 1.12) | 2.42 (1.01, 5.78) | 3.75 (1.95, 7.21) | 2.15 (1.31, 3.51) |
| Siberian weasel | 1.65 (0.57, 4.76) | 0.70 (0.15, 3.34) | 4.09 (1.44, 11.59) | 2.15 (1.06, 4.36) |
| Amur hedgehog | 2.00 (0.52, 7.65) | 9.26 (2.94, 29.15) | 8.95 (3.44, 23.25) | 6.73 (3.15, 14.38) |
| Tolai hare | 2.75 (0.46, 16.40) | 3.92 (1.84, 8.38) | 0.00 (0.00, 0.00) | 2.22 (0.87, 5.68) |

**Appendix S4** Generalized additive mixed model predicting the influence of urban-related and nature-related variables on the population densities of dogs. Model statistics include the degrees of freedom per model (df), logistical likelihood (logLik), Akaike information criterion score corrected for small sample size (AICc), difference in the AICc score from the best-supported model (∆AICc) and explanatory value of each model (AICc weight).

| **Explanatory variables** | **df** | **logLik** | **AICc** | **∆AICc** | **AICc weight** |
| --- | --- | --- | --- | --- | --- |
| RoadDensity(600m) + Pro.Water.body(1600m) | 7 | -75.79 | 169.73 | 0 | 0.24 |
| RoadDensity(600m) + Pro.Cropland(2000m) | 7 | -76.39 | 170.93 | 1.21 | 0.13 |
| RoadDensity(600m) + Pro.Wetlands(1800m) | 7 | -76.56 | 171.26 | 1.53 | 0.11 |
| RoadDensity(600m) + Built(200m) | 7 | -76.70 | 171.55 | 1.82 | 0.10 |
| RoadDensity(600m) + PopuDen(600m) | 7 | -76.73 | 171.62 | 1.89 | 0.09 |
| RoadDensity(600m) + Pro.Forest(200m) | 7 | -76.86 | 171.87 | 2.14 | 0.08 |
| Pro.Water.body(1600m) + Pro.Wetlands(1800m) | 7 | -78.11 | 174.36 | 4.63 | 0.02 |
| Pro.Wetlands(1800m) + Pro.Cropland(2000m) | 7 | -78.12 | 174.40 | 4.67 | 0.02 |
| Pro.Water.body(1600m) + Pro.Cropland(2000m) | 7 | -78.19 | 174.53 | 4.80 | 0.02 |
| Pro.Cropland(2000m) + Built(200m) | 7 | -78.28 | 174.71 | 4.98 | 0.02 |
| Pro.Cropland(2000m) + Pro.Forest(200m) | 7 | -78.40 | 174.94 | 5.21 | 0.02 |
| Pro.Cropland(2000m) + PopuDen(600m) | 7 | -78.40 | 174.96 | 5.23 | 0.02 |
| Pro.Wetlands(1800m) + Pro.Forest(200m) | 7 | -78.51 | 175.16 | 5.43 | 0.02 |
| Pro.Water.body(1600m) + PopuDen(600m) | 7 | -78.67 | 175.49 | 5.76 | 0.01 |
| Pro.Wetlands(1800m) + Built(200m) | 7 | -78.77 | 175.69 | 5.96 | 0.01 |
| Pro.Wetlands(1800m) + PopuDen(600m) | 7 | -78.77 | 175.69 | 5.96 | 0.01 |
| Pro.Forest(200m) + PopuDen(600m) | 7 | -78.84 | 175.83 | 6.10 | 0.01 |
| Pro.Forest(200m) + Pro.Water.body(1600m) | 7 | -78.86 | 175.87 | 6.14 | 0.01 |
| Built(200m) + PopuDen(600m) | 7 | -78.90 | 175.96 | 6.23 | 0.01 |
| Built(200m) + Pro.Forest(200m) | 7 | -79.05 | 176.24 | 6.51 | 0.01 |
| Built(200m) + Pro.Water.body(1600m) | 7 | -79.06 | 176.28 | 6.55 | 0.01 |

**Appendix S5** Generalized additive mixed model predicting the influence of urban-related and nature-related variables on the population densities of cats. Model statistics include the degrees of freedom per model (df), logistical likelihood (logLik), Akaike information criterion score corrected for small sample size (AICc), difference in the AICc score from the best-supported model (∆AICc) and explanatory value of each model (AICc weight).

| **Explanatory variables** | **df** | **logLik** | **AICc** | **∆AICc** | **AICc weight** |
| --- | --- | --- | --- | --- | --- |
| Pro.Wetlands(1000m) + PopuDen(1800m) | 7 | -65.57 | 149.01 | 0 | 0.41 |
| NDVI_400m(400m) + PopuDen(1800m) | 7 | -65.84 | 149.53 | 0.52 | 0.32 |
| PopuDen(1800m) + Pro.Cropland(1600m) | 7 | -66.62 | 151.11 | 2.10 | 0.14 |
| PopuDen(1800m) + Pro.Grassland(1800m) | 7 | -66.74 | 151.34 | 2.33 | 0.13 |
| Pro.Wetlands(1000m) + Pro.Cropland(1600m) | 7 | -70.58 | 159.02 | 10.01 | 0.00 |
| NDVI_400m(400m) + Pro.Cropland(1600m) | 7 | -71.95 | 161.76 | 12.75 | 0.00 |
| NDVI_400m(400m) + Pro.Wetlands(1000m) | 7 | -73.10 | 164.07 | 15.06 | 0.00 |
| Pro.Cropland(1600m) + Pro.Grassland(1800m) | 7 | -73.53 | 164.92 | 15.91 | 0.00 |
| NDVI_400m(400m) + Pro.Grassland(1800m) | 7 | -74.54 | 166.95 | 17.94 | 0.00 |

**Appendix S6** Generalized additive mixed model predicting the influence of urban-related and nature-related variables on the population densities of Siberian weasels. Model statistics include the degrees of freedom per model (df), logistical likelihood (logLik), Akaike information criterion score corrected for small sample size (AICc), difference in the AICc score from the best-supported model (∆AICc) and explanatory value of each model (AICc weight).

| **Explanatory variables** | **df** | **logLik** | **AICc** | **∆AICc** | **AICc weight** |
| --- | --- | --- | --- | --- | --- |
| PopuDen(600m) + Pro.Forest(200m) | 7 | -73.71 | 165.03 | 0 | 0.60 |
| PopuDen(600m) + RoadDensity(200m) | 7 | -75.25 | 168.12 | 3.08 | 0.13 |
| PopuDen(600m) + Pro.Impervious.surfaces(1800m) | 7 | -75.70 | 169.02 | 3.99 | 0.08 |
| PopuDen(600m) + Pro.Cropland(1000m) | 7 | -76.16 | 169.93 | 4.90 | 0.05 |
| PopuDen(600m) + NDVI(1200m) | 7 | -76.35 | 170.31 | 5.28 | 0.04 |
| Pro.Forest(200m) + RoadDensity(200m) | 7 | -76.72 | 171.05 | 6.01 | 0.03 |
| NDVI(1200m) + RoadDensity(200m) | 7 | -76.86 | 171.33 | 6.30 | 0.03 |
| PopuDen(600m) + Pro.Wetlands(1400m) | 7 | -77.29 | 172.19 | 7.16 | 0.02 |
| Pro.Impervious.surfaces(1800m) + RoadDensity(200m) | 7 | -77.73 | 173.08 | 8.05 | 0.01 |
| Pro.Wetlands(1400m) + RoadDensity(200m) | 7 | -77.84 | 173.30 | 8.27 | 0.01 |
| Pro.Cropland(1000m) + RoadDensity(200m) | 7 | -78.63 | 174.88 | 9.84 | 0.00 |
| Pro.Forest(200m) + Pro.Impervious.surfaces(1800m) | 7 | -79.20 | 176.01 | 10.97 | 0.00 |
| NDVI(1200m) + Pro.Impervious.surfaces(1800m) | 7 | -79.91 | 177.44 | 12.41 | 0.00 |
| Pro.Wetlands(1400m) + Pro.Impervious.surfaces(1800m) | 7 | -81.10 | 179.82 | 14.79 | 0.00 |
| Pro.Cropland(1000m) + Pro.Impervious.surfaces(1800m) | 7 | -81.13 | 179.87 | 14.84 | 0.00 |
| Pro.Forest(200m) + Pro.Wetlands(1400m) | 7 | -82.18 | 181.98 | 16.95 | 0.00 |
| Pro.Forest(200m) + NDVI(1200m) | 7 | -83.14 | 183.89 | 18.85 | 0.00 |
| NDVI(1200m) + Pro.Wetlands(1400m) | 7 | -83.31 | 184.23 | 19.20 | 0.00 |
| Pro.Cropland(1000m) + Pro.Wetlands(1400m) | 7 | -83.33 | 184.27 | 19.24 | 0.00 |
| Pro.Forest(200m) + Pro.Cropland(1000m) | 7 | -83.42 | 184.45 | 19.42 | 0.00 |
| Pro.Cropland(1000m) + NDVI(1200m) | 7 | -83.84 | 185.29 | 20.26 | 0.00 |

**Appendix S7** Generalized additive mixed model predicting the influence of urban-related and nature-related variables on the population densities of Amur hedgehogs. Model statistics include the degrees of freedom per model (df), logistical likelihood (logLik), Akaike information criterion score corrected for small sample size (AICc), difference in the AICc score from the best-supported model (∆AICc) and explanatory value of each model (AICc weight).

| **Explanatory variables** | **df** | **logLik** | **AICc** | **∆AICc** | **AICc weight** |
| --- | --- | --- | --- | --- | --- |
| Pro.Grassland(200m) + Pro.Impervious.surfaces(2000m) | 7 | -93.35 | 205.00 | 0 | 0.94 |
| Pro.Grassland(200m) + PopuDen(600m) | 7 | -96.66 | 211.64 | 6.63 | 0.03 |
| PopuDen(600m) + Pro.Wetlands(800m) | 7 | -98.44 | 215.18 | 10.18 | 0.01 |
| NDVI(600m) + PopuDen(600m) | 7 | -98.53 | 215.37 | 10.37 | 0.01 |
| Pro.Wetlands(800m) + Pro.Impervious.surfaces(2000m) | 7 | -99.08 | 216.46 | 11.46 | 0.00 |
| PopuDen(600m) + Pro.Cropland(1800m) | 7 | -99.15 | 216.60 | 11.60 | 0.00 |
| NDVI(600m) + Pro.Impervious.surfaces(2000m) | 7 | -99.42 | 217.14 | 12.14 | 0.00 |
| Pro.Cropland(1800m) + Pro.Impervious.surfaces(2000m) | 7 | -99.54 | 217.39 | 12.38 | 0.00 |
| Pro.Grassland(200m) + Pro.Wetlands(800m) | 7 | -100.39 | 219.10 | 14.09 | 0.00 |
| Pro.Grassland(200m) + Pro.Cropland(1800m) | 7 | -100.85 | 220.01 | 15.00 | 0.00 |
| Pro.Grassland(200m) + NDVI(600m) | 7 | -100.85 | 220.01 | 15.01 | 0.00 |
| Pro.Wetlands(800m) + Pro.Cropland(1800m) | 7 | -102.98 | 224.26 | 19.26 | 0.00 |
| NDVI(600m) + Pro.Wetlands(800m) | 7 | -103.45 | 225.21 | 20.21 | 0.00 |
| NDVI(600m) + Pro.Cropland(1800m) | 7 | -103.46 | 225.22 | 20.22 | 0.00 |

**Appendix S8** Generalized additive mixed model predicting the influence of urban-related and nature-related variables on the population densities of Tolai hares. Model statistics include the degrees of freedom per model (df), logistical likelihood (logLik), Akaike information criterion score corrected for small sample size (AICc), difference in the AICc score from the best-supported model (∆AICc) and explanatory value of each model (AICc weight).

| **Explanatory variables** | **df** | **logLik** | **AICc** | **∆AICc** | **AICc weight** |
| --- | --- | --- | --- | --- | --- |
| Pro.Grassland(1600m) + RoadDensity(200m) | 7 | -49.79 | 118.67 | 0 | 0.21 |
| Pro.Grassland(1600m) + Pro.Cropland(600m) | 7 | -49.94 | 118.96 | 0.29 | 0.18 |
| NDVI(1600m) + Pro.Cropland(600m) | 7 | -50.53 | 120.15 | 1.48 | 0.10 |
| RoadDensity(200m) + Pro.Cropland(600m) | 7 | -50.64 | 120.37 | 1.70 | 0.09 |
| Pro.Wetlands(200m) + Pro.Grassland(1600m) | 7 | -50.66 | 120.42 | 1.75 | 0.09 |
| NDVI(1600m) + Pro.Grassland(1600m) | 7 | -50.72 | 120.53 | 1.86 | 0.08 |
| Pro.Wetlands(200m) + RoadDensity(200m) | 7 | -50.85 | 120.79 | 2.12 | 0.07 |
| NDVI(1600m) + RoadDensity(200m) | 7 | -50.86 | 120.82 | 2.15 | 0.07 |
| Pro.Cropland(600m) + Pro.Wetlands(200m) | 7 | -50.88 | 120.85 | 2.18 | 0.07 |
| Pro.Wetlands(200m) + NDVI(1600m) | 7 | -51.35 | 121.79 | 3.13 | 0.04 |
